# A Red-Blooded Transcription Factor

**DOI:** 10.1371/journal.pbio.0020282

**Published:** 2004-08-17

Every multicellular organism depends on the coordinated actions of a multitude of cell types, each designed to carry out specific jobs. In vertebrates, for example, the task of ferrying oxygen to organs, tissues, and cells throughout the body is shouldered by red blood cells. These cells must develop early in the embryo to nourish the body and must be maintained at the right levels throughout adulthood to keep the organism healthy.

Specialized red blood cells arise from undifferentiated stem cells in a developmental process called hematopoiesis. All vertebrates have the same basic plan for hematopoiesis during their development, with one group of stem cells producing embryonic blood cells and, later, another group in a different part of the body making adult blood cells. Such developmental programs are tightly orchestrated by suites of transcription factors—proteins that turn genes on and off.

But which transcription factors act—and how and where they act—is still largely unknown. Scientists and doctors alike want to better understand differentiation in order to grasp not only how it proceeds normally but also how it can go wrong and lead to diseases, such as leukemia, in which white blood cells become cancerous, and aplastic anemia, in which bone marrow stem cells make too few red blood cells.

To discover the genes involved in hematopoiesis, Leonard Zon's group at Children's Hospital, Boston, and their collaborators exposed zebrafish to mutagens and then studied the individuals that developed relevant disorders. In 1996, the group produced three zebrafish lines with embryos lacking fully differentiated red blood cells; since these fish also had especially shimmery tails, the lines were named moonshine.

Now Zon and his colleagues have identified the mutant gene responsible for the moonshine zebrafish lines' peculiar traits. The *moonshine (mon)* gene, it turns out, encodes a transcription factor with wide effects on the embryo's mesoderm, the set of cells that eventually form the circulatory system, muscles, and skeleton. The researchers found that all three lines of mutant zebrafish had mutations in the *mon* gene.

In the mutant fish, stem cells that produce red blood cells were present initially, but the defective moonshine protein was unable to keep the blood cells alive. These blood cells underwent apoptosis, or programmed cell death, leaving the fish unable to make red blood cells, and they died at about two weeks old.[Fig pbio-0020282-g001]

**Figure pbio-0020282-g001:**
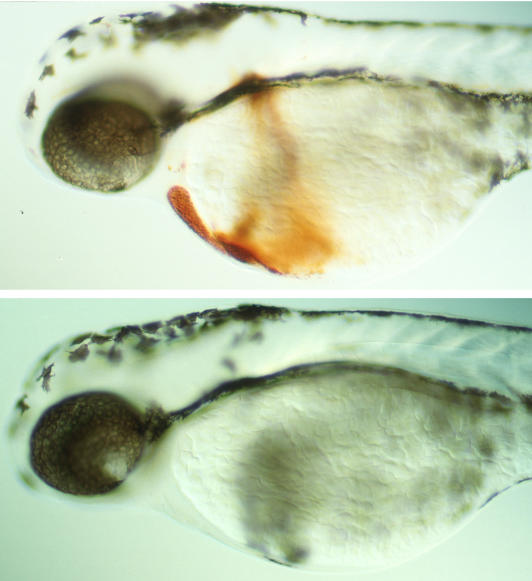
Hemoglobin staining shows that hematopoiesis is defective in a *moonshine* mutant (bottom) compared to a wild-type zebrafish embryo (top)

The researchers found that the protein encoded by the *mon* gene is most similar to the human and mouse Tif1-gamma, one of a family of proteins known to link DNAbinding proteins with other factors that activate or repress gene activity. Using a DNA analysis technique in mouse cell cultures, the researchers found Tif1-gamma in nuclear bodies, multi-protein complexes in cell nuclei that help regulate gene expression. But the Tif1-gamma nuclear bodies didn't fit into any known class of such complexes, so it's an open question how Tif1-gamma acts and what other proteins help regulate it. Finding a transcription factor such as Tif1-gamma involved in early cell specialization opens a door to a suite of studies on the targets of the transcription factor, as well as other genes that act along with *mon* to affect red blood cell development.

